# Host-Specific Phenotypic Plasticity of the Turtle Barnacle *Chelonibia testudinaria*: A Widespread Generalist Rather than a Specialist

**DOI:** 10.1371/journal.pone.0057592

**Published:** 2013-03-01

**Authors:** Chi Chiu Cheang, Ling Ming Tsang, Ka Hou Chu, I-Jiunn Cheng, Benny K. K. Chan

**Affiliations:** 1 Biodiversity Research Center, Academia Sinica, Taipei, Taiwan; 2 Simon F. S. Li Marine Science Laboratory, School of Life Sciences, The Chinese University of Hong Kong, Shatin, Hong Kong; 3 Institute of Marine Biology, National Taiwan Ocean University, Keelung, Taiwan; Ecole Normale Supérieure de Lyon, France

## Abstract

Turtle barnacles are common epibionts on marine organisms. *Chelonibia testudinaria* is specific on marine turtles whereas *C. patula* is a host generalist, but rarely found on turtles. It has been questioned why *C. patula*, being abundant on a variety of live substrata, is almost absent from turtles. We evaluated the genetic (mitochondrial COI, 16S and 12S rRNA, and amplified fragment length polymorphism (AFLP)) and morphological differentiation of *C. testudinaia* and *C. patula* from different hosts, to determine the mode of adaptation exhibited by *Chelonibia* species on different hosts. The two taxa demonstrate clear differences in shell morphology and length of 4–6^th^ cirri, but very similar in arthropodal characters. Moreover, we detected no genetic differentiation in mitochondrial DNA and AFLP analyses. Outlier detection infers insignificant selection across loci investigated. Based on combined morphological and molecular evidence, we proposed that *C. testudinaria* and *C. patula* are conspecific, and the two morphs with contrasting shell morphologies and cirral length found on different host are predominantly shaped by developmental plasticity in response to environmental setting on different hosts. *Chelonibia testudinaria* is, thus, a successful general epibiotic fouler and the phenotypic responses postulated can increase the fitness of the animals when they attach on hosts with contrasting life-styles.

## Introduction

**Figure 1:**
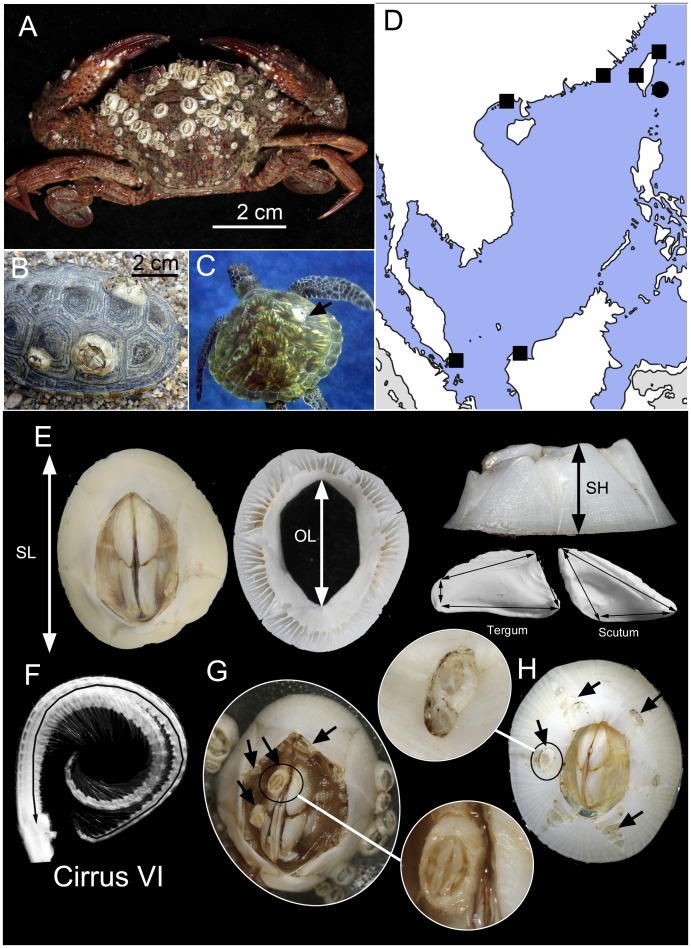
A. *Chelonibia patula* is commonly epibiotic on crustaceans surfaces. B., C. *Chelonibia testudinaria* (indicated by black arrow in C) is common on shell surface of marine turtles. D. Sampling locations of *C. patula* (squares) and *C. testudinaria* (circle) in the present study. E. Shell parameters measured for morphological analysis. SL – shell length, OL – orifice length, SH – shell height, ST – shell thickness. F. Cirrus IV of *C. testudinaria*, showing the length measured for morphological analysis. Cirri V and VI not shown due to similarity in morphology. G. *Chelonibia patula*, showing the small dwarf males (indicated by arrows) settled randomly on shell surface and orifice opening. H. *Chelonibia testudinaria*, showing the dwarf males (indicated by arrows) settled on the oval depression in the radii of the shell plates.

Barnacles of the superfamily Coronunloidea are epibionts on a range of marine organisms including whales and turtles. Most of the species are specialists restricted to one or a few hosts [1], [2]. The turtle barnacle *Chelonibia testudinaria* (Linnaeus, 1758) has long been known to inhibit predominantly on the carapace, flippers and skin of marine turtles [3]. It occurs in high abundance on the loggerhead turtle *Caretta caretta* (Linnaeus, 1758) [4], but is rarely found on other animals (but see [5], [6]). In contrast, the cogeneric species, *Chelonibia patula* (Ranzani, 1818) that exhibits distinct shell morphology (Figures 1G, H; [7]), is a generalist, occurring on a wide variety of hosts, including decapods, gastropods, stomatopods and even sea snakes, but rarely observed on turtles (Figure 1A–C; [1], [8]). It has been questioned why *C. patula*, which is able to survive on such a broad range of hosts, is almost absent from marine turtles [7]; but see [9], [10].

One possible scenario is the heterogeneous environment of the turtle host and other animals exert strong selection on the barnacle individuals settle on it. Any individuals with sub-optimal fitness will be selected against which would preclude gene flow between populations on turtle and other animals. This would drive adaptive divergence, resulting in inheritable morphological traits (e.g. shell morphology) and ultimately speciation [11], [12], [13]. Speciation by differential host adaptation under similar scenario is widely observed in other symbiotic barnacles (e.g. [14], [15], [16]). On the other hand, if the selection pressure is more gentle (i.e. sub-lethal), reproductive isolation between populations could not be achieved. The species could either adapt by differential divergence within genome of which genetic divergence is only observed across loci under selection while genetic homogeneity in other neutral loci is maintained by continuous gene flow [17], [18], or by phenotypic plasticity in response to different environmental settings [19]. In either of the latter two circumstances, reproductive barrier is absent between the two “species”. The morphological differences are determined by a few loci under selection or induced by environmental cues, and they should be considered conspecific. Which of the routes the organisms is selected towards depends on various factors, such as the strength of selection, the cost of development of adaptive divergence and level of gene flow. For instance, phenotypic plasticity is favored when the environmental changes are rapid and/or environments are spatially or temporally heterogeneous [20], [21]. Which form of adaptation has developed in the two *Chelonibia* species adapting to the different hosts remains unclear.

To understand whether *Chelonibia testudinaria* and *C. patula* exhibit host specific adaptation through speciation, differential divergence in genome or phenotypic plasticity, investigation on the genetic divergence pattern between the two species is the prerequisite. Here, we attempted to determine the mode of adaptation in the two taxa using an integrated genetic (three genes COI, 12S and 16S rRNA in the mitochondrial genome plus AFLP) and morphological (shell, opercular plates and arthropodal characters) analyses on *Chenolibia testudinaria* and *C. patula* populations from different hosts and geographic locations.

Mitochondrial markers, on one hand, have been proven to be useful tools to delineate barnacle species due to their fast evolving nature and are commonly applied in barnacle phylogenetic and population genetic studies [22], [23], [24]. AFLP, on the other hand, is a genome scanning technique that not only assesses the genome wide differentiation but also identifies any outlier loci under selection pressure [25] and this technique has been applied in barnacle population studies previously [26]. Moreover, by comparing the genetic profile with a quantitative morphological assessment of the two species, we can determine correlation between genetic and morphological divergence and thus, identify the possible mode of adaptation [27], [28]. Coherence between divergence of the genetic profile and the morphology of the two species would suggest occurrence of adaptive divergence, whilst the disassociation of the two measurements may reflect the predominance of phenotypic plasticity (i.e. morphological difference with no distinctive genomic difference). Together, these data provide invaluable information on genetic and morphological responses of marine animals in adapting to spatially heterogeneous environments, i.e. different epibiotic hosts, in nature.

## Results

### Morphological Investigation

**Figure 2:**
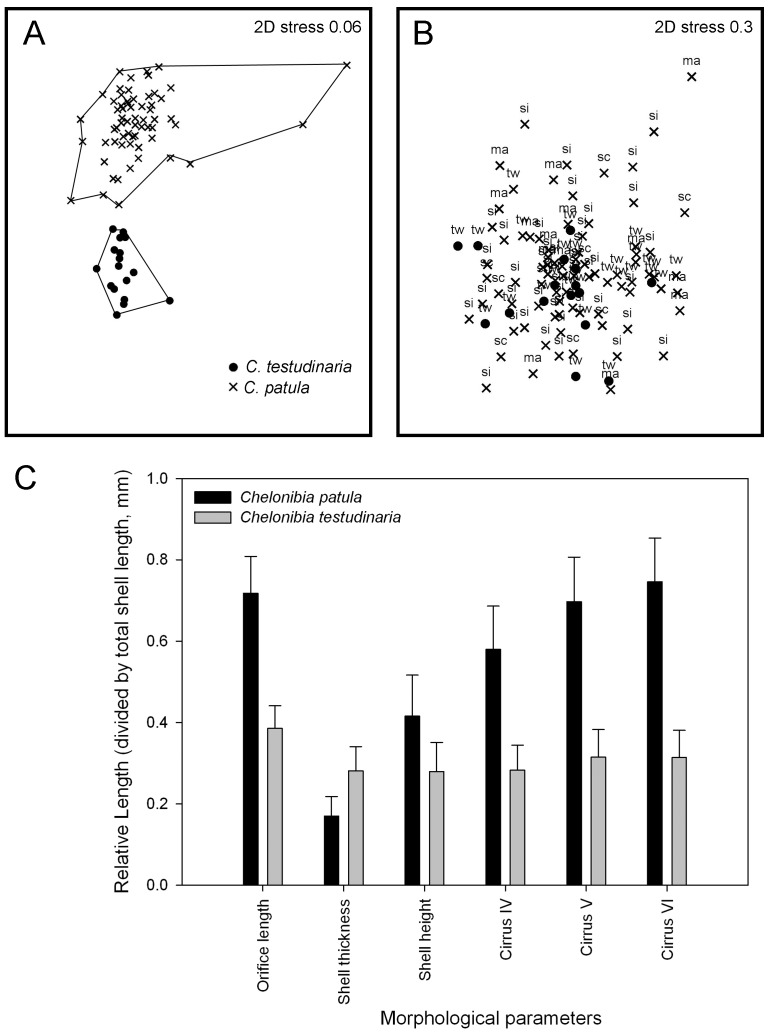
A. nMDS plots showing ordinations of the morphological characters of *Chelonibia patula* and *C. testudinaria*. B. nMDS plots showing the AFLP analysis of *C. patula* and *C. testudinaria*. Refer to table 2 for the acronyms of the population. C. Mean (± SD), of the shell parameters and cirrus length (relative to the total shell length) of *C. patula* and *C. testudinaria*.

The external shell of *C. patula* is conical and with smooth surface (Figure 1E, G). Small sized individuals which are dwarf males often attached randomly on the shell surface and orifice openings (Figure 1G). *Chelonibia testudinaria* was lower in profile instead of conical, with oval depressions on the radii, which are the junctions between the shell plates. Small dwarf males were often found settled in those oval depressions (Figure 1H). From multivariate nMDS plots on shell and arthropodal characters, the ordinations of *C. testudinaria* and *C. patula* were separated into two distinct clusters (Figure 2A). The nMDS plot with low 2D stress value of 0.06 indicated that the clusters were well separated (Figure 2A). From ANOSIM analysis, the morphological characters of *C. patula* and *C. testudinaria* were significantly different (R = 0.85, *P*<0.05). From SIMPER analysis, the most significant characters (>10% contributed differences) were the length of the cirri IV, V and VI and the opercular length. *Chelonibia patula* had a relatively larger orifice length and cirri IV, V and VI were almost twice as long as *C. testudinaria* (Figure 2C).

### Arthropodal Characters

**Figure 3:**
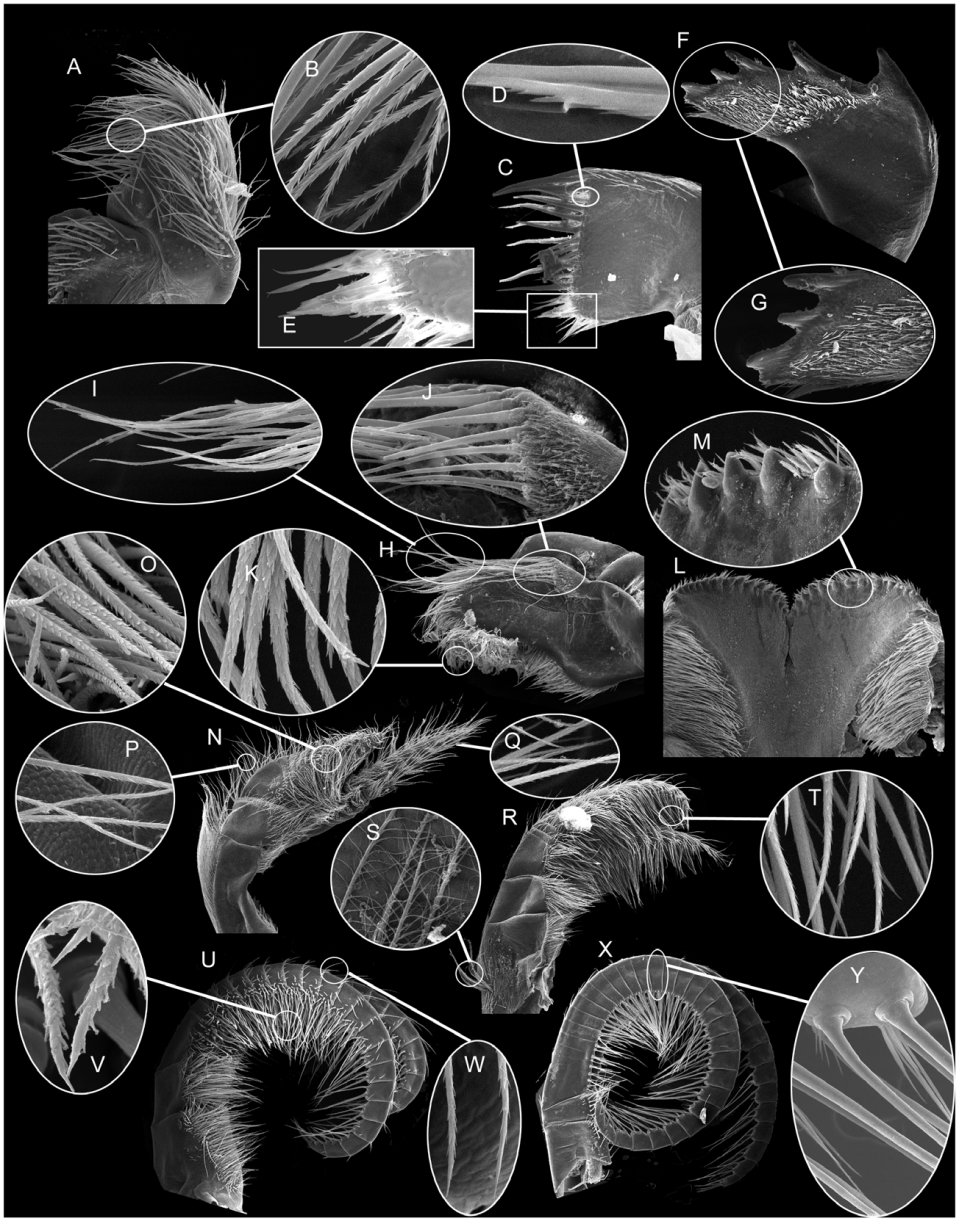
Scanning electron microscopy of mouth parts and cirri of *Chelonibia testudinaria*. A. Maxilla, B. Serrulate setae on maxilla. C. Maxillule, D. Serrulate setae on maxillule. E. Setae on cutting edge of maxillule. F. Mandibles, G. 3^rd^ –5^th^ teeth and the lower margin of mandible. H. Mandibular palp, showing simple type setae on the inferior margin (I, J) and serrulate setae on superior margin (K). L. Labrum showing enlarged view of teeth on cutting edge (M). N. cirrus I, showing densely pectinated serrulate setae (O) and serrulate setae on rami (P, Q). R. Cirrus II, with serrulate setae (T) and pappose setae (S). U. Cirrus III, with serrulate type setae (V, W) on rami. X. Cirrus VI, showing the intermediate segment (Y).

From SEM, the structure of mouth parts and setal types of the cirri were similar between *C. patula* and *C. testudinaria*. The maxilla was composed of fine serrulate setae (Figure 3A, B). The maxillule was not notched, with serrulate setae on the cutting edge (Figure 3C, D, E). The mandible consisted of five teeth, with second and third teeth bi-dentate. The lower margin was short and composed of several spines (Figure 3F, G). The mandibular palp consisted of two types of setae, with serrulate setae on the superior margin and simple setae on the inferior margin (Figure 3H, I, J, K). Mandibles were strongly notched, with an array of large sharp teeth (Figure 3L, M). In cirrus I, the posterior rami were longer than the anterior rami, both with densely pectinated serrulate setae (Figure 3N, O, P, Q). In cirrus II, the anterior and posterior rami were similar in length, both with fine serrulate setae (Figure 3R, T) and the basipod with pappose setae (Figure 3S). Both rami of cirrus III were equipped with serrulate type setae (Figure 3U, V, W). Cirri IV to VI were similar in length and morphology, with each segment having two pairs of long serrulate setae and two pairs of short simple setae (Figure 3X, Y).

### Mitochondrial DNA Markers

**Figure 4:**
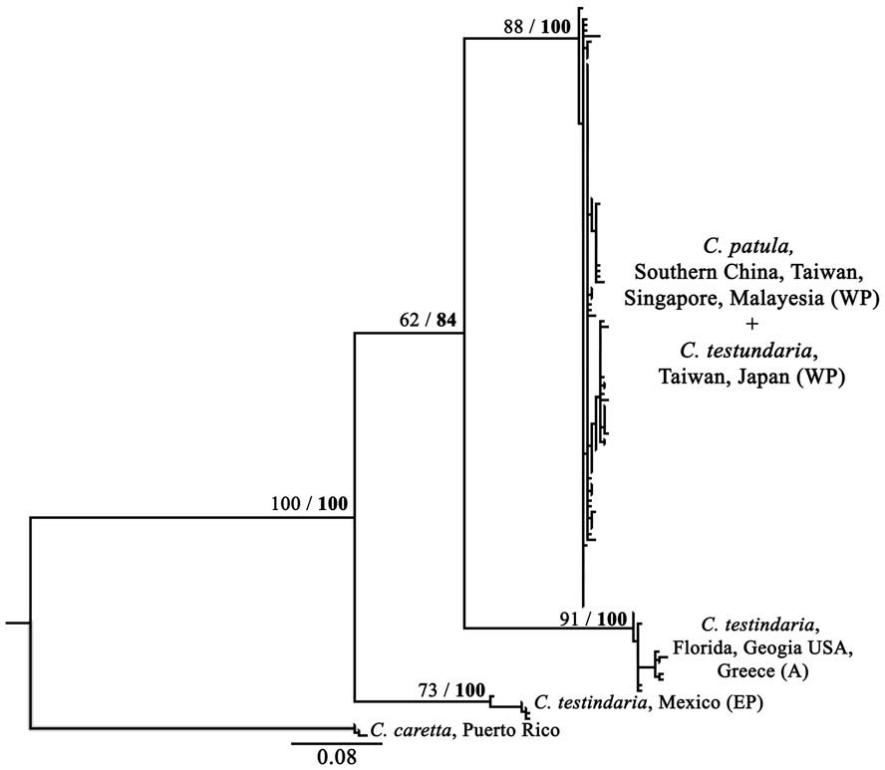
Maximum likelihood (ML) tree of *Chelonibia testudinaria* and *C. patula* in this study with sequences of *C. testudinaria* from Rawson et al. (2003) (GenBank accession nos.: AY174312–16, 24–28, 34–8, 42–46, 58–62) and outgroup *C. caretta* (FJ385728-30). ML and Bayesian inference (BI) analyses yield the same topology. Bootstrap (1,000 replicates) values for ML (normal) and the posterior probabilities for BI (bold) analyses are indicated at the nodes. (EP: eastern Pacific; WP: western Pacific; A: Atlantic).

**Figure 5:**
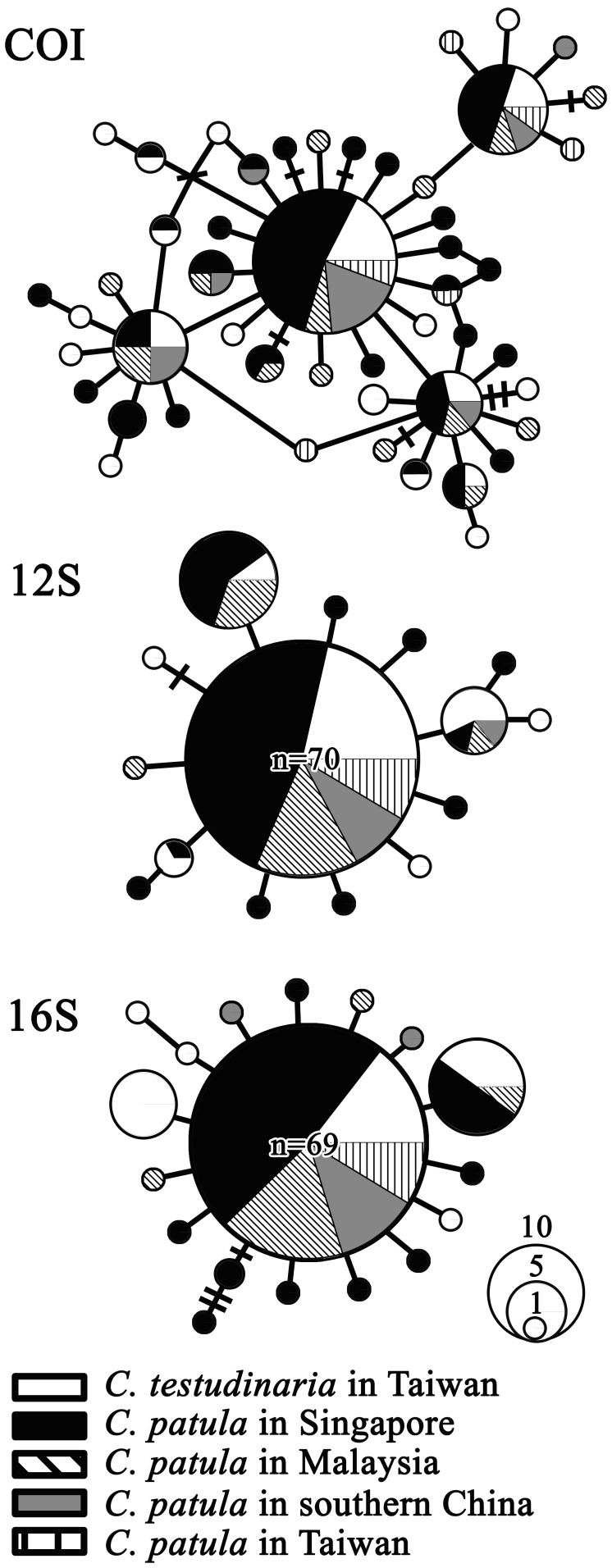
TCS network of *Chelonibia testudinaria* (white portion) and *C. patula* (black and hatched portions) based on mitochondrial COI, 12S and 16S rRNA markers. Locality of populations of *C. patula* is indicated in respective black and hatched portions.

*Chelonibia testudinaria* from the present study, together with those from the Pacific coast of Japan [29], clustered with *C. patula* (Figure 4). The K2P distances between these three groups were between 0.0042 and 0.0065 (Table 1). Populations of *C. testudinaria* in the Atlantic Ocean formed a sister clade to the western Pacific populations (Figure 4) and demonstrated K2P distances of 0.1057–0.1066 (Table 1). The eastern Pacific is the most divergent population of *C. testudinaria* (Figure 4), with K2P distance up to 0.1210 from the other populations (Table 1). Haplotypes within each of the three mitochondrial markers revealed by the TCS analysis did not group together according to species nor locality of populations (Figure 5). All dominant haplotypes could be found in both species as well as in different populations (Figure 5).

**Table 1:** Kimura-2-parameter distance among outgroup *Chelonibia caretta*, the five populations of *C. testudinaria* in Rawson et al. *(*2003) as well as *C. testudinaria* and *C. patula* examined in this study based on COI.

	CP	CT	CT (WP)	CT (A)	CT (EP)
CP					
CT	0.0046				
CT (WP)	0.0055	0.0056			
CT (A)	0.1066	0.1057	0.1059		
CT (EP)	0.1225	0.1217	0.1216	0.121	
CC	0.1932	0.1928	0.1925	0.1885	0.1997

Note: CC, *C. caretta*; CP, *C. patula*; CT, *C. testudinaria*; (A), Atlantic; (EP), eastern Pacific; (WP), western Pacific.

Both Nei’s genetic and nucleotide diversities of *C. testudinaria* and *C. patula* were highest for the COI dataset (Table 2), while those from 16S and 12S rRNA were relatively low (see values in Table 2). The differences of the diversity indexes between the two species were not significant (unpaired t test; *P>*0.05) for all three mitochondrial markers, except the Nei’s genetic diversity based on 16S rRNA (unpaired t test; *P<*0.05). The results of AMOVA demonstrated that the “between species” variances were, whether significant or not, small for all three markers. Most of the variance occurred at the “within population” level (Table 3).

**Table 2:** Genetic diversity (mean ± standard error) of *Chelonibia testudinaria* and *C. patula* based on mitochondrial COI, 16S, and 12S rRNA markers and AFLP.

	COI (642 bp)			16S (503–506 bp)			12S (302–303 bp)			AFLP	
	n (n_h_)	*H*	π	n (n_h_)	*H*	π	n (n_h_)	*H*	π	n	*H*
*C. testudinaria*											
Taiwan	25 (19)	0.98±0.02	0.0049±0.0029	24 (6)	0.74±0.06	0.0021±0.0016	25 (7)	0.63±0.10	0.0029±0.0024	15	0.336±0.008
Whole species	25 (19)	0.98±0.02	0.0049±0.0029	24 (6)	0.74±0.06	0.0021±0.0016	25 (7)	0.63±0.10	0.0029±0.0024	15	0.336±0.008
*C. patula*											
Southern China(sc)	10 (7)	0.91±0.08	0.0029±0.0021	10 (3)	0.38±0.18	0.0008±0.0009	7 (2)	0.29±0.20	0.0009±0.0013	6	0.372±0.008
Taiwan (tw)	6 (6)	1.00±0.10	0.0045±0.0031	6 (1)	0.00±0.00	0.0000±0.0000	6 (1)	0.00±0.00	0.0000±0.0000	3	0.365±0.009
Malaysia (ma)	15 (14)	0.99±0.03	0.0046±0.0029	15 (4)	0.37±0.15	0.0008±0.0009	15 (4)	0.54±0.13	0.0020±0.0019	7	0.356±0.008
Singapore (si)	47 (27)	0.95±0.02	0.0039±0.0024	47 (10)	0.50±0.09	0.0016±0.0013	48 (11)	0.52±0.08	0.0022±0.0019	45	0.330±0.008
Whole species	78 (38)	0.95±0.01	0.0039±0.0024	78 (14)	0.42±0.07	0.0012±0.0011	76 (12)	0.47±0.07	0.0019±0.0017	61	0.332±0.008
Total	103 (49)	0.95±0.01	0.0042±0.0025	102 (16)	0.42±0.06	0.0012±0.0011	101 (15)	0.51±0.06	0.0022±0.0019	76	0.334±0.003

Noted there is not statistical significance for all the diversities between the two species except with the Nei’s genetic diversity estimated for 16S rRNA. Acronyms are shown in brackets besides the respective populations.

Note: n, number of samples; n_h_, number of haplotypes; *H*, Nei’s genetic diversity; π, nucleotide diversity.

**Table 3:** Global locus-by-locus analyses of molecular variance (AMOVA) results as weighted averages over loci.

Marker	Source of Variance	% of variation	Φ	*p*
COI	Between species	1.17	1.554	0.360^a^
	Within species, amongpopulations	−1.05	3.379	0.660^a^
	Within population	99.88	131.067	0.633^b^
16S rRNA	Between species	12.62	1.994	0.010^a^
	Within species, amongpopulations	−2.46	0.647	0.887^a^
	Within population	89.84	35.535	0.011^b^
12S rRNA	Between species	5.62	0.725	0.003^a^
	Within species, amongpopulations	−3.46	0.504	0.893^a^
	Within population	97.84	31.701	0.521^b^
AFLP	Between species	−1.21	75.772	0.894^a^
	Within species, amongpopulations	3.10	223.297	>0.001^a^
	Within population	98.11	4140.206	>0.001^b^

Note: ^a^Random value larger than or equal to the observed value;

b Random value smaller than or equal to the observed value.

### AFLP Pattern

**Figure 6:**
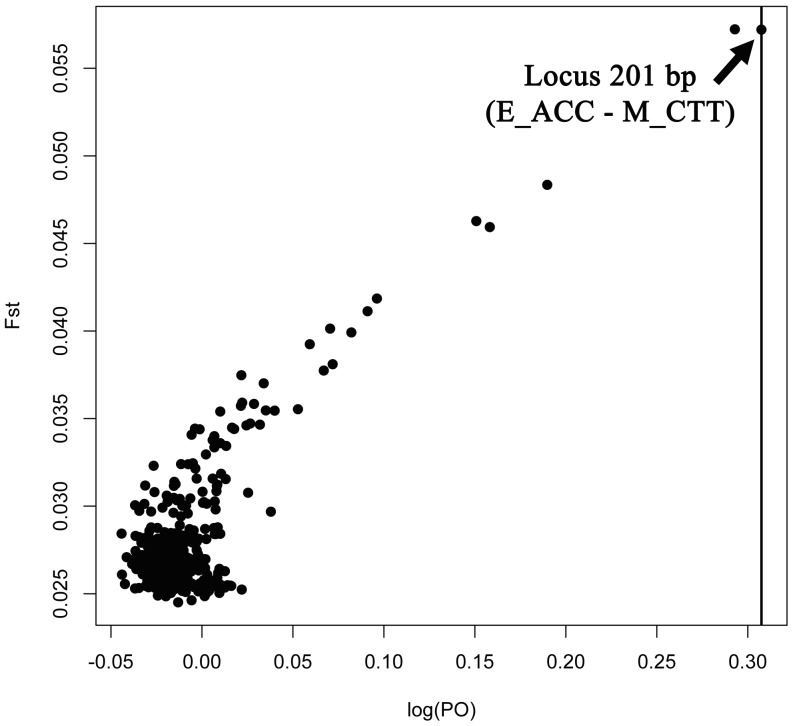
F_ST_ value for each of the AFLP loci and their associated posterior odds (PO). Solid vertical line represents the threshold value (false discovery rate of 0.05) of PO; loci with PO larger than the threshold regarded as outliers. Note that PO is equivalent to Bayes Factors when the prior odds are set to 1 (refer to text for details).

While 120 AFLP bands on average (<200 bands) were scored for each primer combination in the present study, the biases on the outlier identification introduced by size homoplasy of AFLP was assumed to be small (see [30]). A total of 340 polymorphic loci were detected from the AFLP pattern generated. The number of polymorphic loci for *C. testudinaria* and *C. patula* was 321 (94.4%) and 308 (90.6%) respectively. The Nei’s genetic distance (expected heterozygosity) estimate based on AFLP ranged from 0.33 to 0.37 (Table 2). There was no significant difference between the two species in terms of both Nei’s genetic diversity (unpaired t test; *P>*0.05) and pairwise distance calculated based on allelic frequency (permutation test in AFLPsurv; F_ST_ = 0.0012, *p* (high) >0.05; nMDS ordination Figure 2C). The results of AMOVA from the AFLP analysis were similar to those from the mitochondrial markers, showing a majority of “within population” variance (Table 3). One locus (201 bp of combination E_ACC/M_CTT) out of 340 loci (0.29%) was identified to possess a F_ST_ value significantly higher than that across all loci (F_ST_ = 0.06, Alpha = 0.66, Log_10_(PO) = 0.31), when the FDR was set at 0.05 (Figure 6). This locus was inferred to be under selection pressure.

## Discussion

### Phylogenetic Relationship and Host Specificity of *Chelonibia testudinaria*/*C*. *patula*

*Chelonibia patula* and *C. testudinaria* exhibit distinct morphological differences in external shell characters and length of cirri. However, the arthropodal characters, which are important characters in species identification, are indistinguishable between the two species. Furthermore, the two species do not exhibit significant differentiation in mitochondrial gene sequences nor AFLP genotypes. We found little evidence to support the species separation of the two taxa and we therefore, propose that *Chelonibia patula* (Ranzani, 1818) is a junior synonym to *C. testudinaria* (Linnaeus, 1758). *C. testudinaria*/*C. patula* should be regarded as a host generalist live broadly on sea turtles, decapods, gastropods and even sea snakes [1], [8], instead of an obligate epibiont previously believed.

*C. testudinaria* constitutes of three major lineages in the world’s oceans, the eastern Pacific, western Pacific and Atlantic (including the Mediterranean) based on the divergence pattern in mitochondrial COI [29]. In the present study, all *C. testudinaria* and *C. patula* collected in SE Asia and Taiwan belong to the western Pacific group (collected from Japan in [29]). High gene flow was observed among populations collected from different locations and hosts despite that *C. testudinaria* completes its larval development in only about nine days (six naupliar and one cypris stages [31]). Provided the species could survive and settle on such a broad range of animals, dispersal of any of these hosts on which the barnacles attach could result in transport of barnacles and homogenize the genetic composition among populations. This corroborates previous finding that host migration facilitates long distance dispersal of *Chenobia*, while phylogeographic structure only occurs across ocean basins that impact host migration [29].

### Host Specific Adaptation via Phenotypic Plasticity

The lack of genetic differentiation between the *Chelonibia* populations on turtles and benthic crustaceans rejects the hypothesis that shell morphology differentiation is a result of host associated speciation. Hence, the subsequent question is whether the observed differences in shell morphology is a result of differential selection on some of the loci that are responsible for shell development in spite of high homogeneity in neutral loci in the genome genotypes, or phenotypic plasticity in development, i.e. genotype by environmental interactions [32].

In the present study, only one out of the >300 AFLP loci (0.29% of total) is significantly deviated from neutral expectation. This value appears to be significantly smaller than those reported in other studies using similar approaches [31], [32]. For instance, the best investigated case of differential selection on genome between two morphs of the periwinkle *Littorina saxatilis* (Olivi, 1792), induced by physical environments and predation pressure, revealed ∼5% of AFLP loci exhibiting significantly higher level of differentiation than expected under a neutral model [33], [34]. Recent study on the killifish *Fundulus heteroclitus* (Linnaeus, 1766) detected a smaller number of SNP loci, 1.4–2.5%, that are possibly influenced by selection in the population inhabiting polluted areas [35]. Therefore, the role of selection in generating the shell morphology divergence among the barnacles inhabiting turtle and other hosts is insignificant, if any. Definitely, we cannot eliminate the possibilities that genotypes controlling differential preference in settlement on certain host (e.g. turtle vs. crab) remain unidentified; or the single outlier locus could represent a mutation on a gene responsible for host selection or transcriptional regulation could cause the critical differences between two species in adapting the environment based on the present data. It is a more parsimonious explanation that the distinct shell morphology represents phenotypic response, instead of selection for particular genotypes.

Phenotypic plasticity may allow the organisms to rapidly adapt to new environment of which adaptive divergence is usually too slow to develop [21], [36]. Hence, phenotypic plasticity is expected to allow the turtle barnacles to switch from host to host in a single generation, i.e. the larvae of a barnacle living on turtle can possibly recruit on decapods, and vice versa. This would be more beneficial for adopting phenotypic plasticity over adaptive divergence in such a variable environmental setting, in particular the gene flow is inferred to be high that would hamper divergence as well. Moreover, the high genetic diversity in the species shown in the present study also provides plenty of raw materials for the development of phenotypic plasticity. It is widely acknowledged that intertidal acorn barnacles experiencing variation in predation pressure and wave action develop different shell forms and lengths of cirri [37], [38], [39], [40], [41]. Thus the development of barnacle shell is apparently rather plastic in nature. *Chelonibia testudinaria* on turtles examined in the present study have a much depressed shell, and the radii between the shell plates have oval-shaped depressions that can house dwarf males [31]. Most of barnacle species are hermaphroditic, especially the intertidal species which live in dense population. In some species where the mating group size is small and patchy, these species often have dwarf males attached on the large hermaphrodites or females to facilitate mating success. *C. testudinaria* living on marine turtle are patchily and sparely distributed. The presence of dwarf males in those oval-shaped depressions of external shells can increase the mating success. On the contrary, *Chelonibia* living on benthic crustaceans have a higher shell with larger orifice and the radii between the shell plates are smooth. In general, *Chelonibia* barnacles on turtles are relatively larger than those on benthic crustaceans. Such difference in size is likely due to moulting of the host crustaceans. After moulting the epibiotic barnacles would be removed together with the old exoskeleton. Adult *Portunus pelagicus* (Linnaeus, 1758), for example, moults every 2–3 weeks [42]. The carapace scutes of marine turtles probably shed at a much slower rate as their growth rate is very slow (1–2 cm in carapace length per year; [43]), allowing the barnacles to grow larger. As a result, the size difference between the *Chelonibia* populations on turtles and crustaceans may be related to age rather than phenotypic responses. In contrast, morphological characters such as the pits on the shell plates, are found on specimens from the turtles and crustaceans of similar size (see Figure 1) and hence unlikely to be size or age dependent.

Differences in the substratum and physical stress on the surfaces of marine turtles and benthic crustaceans may also be responsible for phenotypic responses. The carapace of crustaceans is chitinous in nature [44] whilst the scutes on marine turtles are made up of keratin [45]. Benthic marine crustaceans stay consistently at a depth of 20–40 m (see [46]). Marine turtles such as *Chelonia mydas* (Linnaeus, 1758) can dive to depth of 10 m or more and can stay submerged for about 20 minutes before swimming back to the surface for a short breath [47]. Barnacles living on marine turtles may experience frequent hydrostatic pressure changes due to the ascending and descending movements of the turtles. Other than frequent changes in hydrostatic pressure and exposure to air, the major environmental difference between the surface of marine turtles and benthic crustaceans is flow velocity, as turtles exhibit much higher swimming speed (about 0.5 m s^−1^ for *Chelonia mydas* [47]) than benthic crustaceans. A barnacle shell of lower profile in *Chelonibia* living on marine turtles can reduce drag under strong currents. The pits in the shell radii may facilitate the settlement of cyprids and development of dwarf males in a rapid flow environment (Figure 1H). This may also explain the shorter cirri IV to VI of *Chelonibia* on turtles compared to the cirri of those on benthic crustaceans as the cirral lengths are usually negatively correlated to wave action and water flow in surrounding environment (see [39], [41]). Admittedly, these potential explanations remain speculative without further evidence from laboratory experiments or field observation. “Common garden” [48] or reciprocal transplant experiments [49] are needed to determine the actual environmental factors that shape the shell morphology of the turtle barnacles. However, larval culture, induced settlement and long term cultivation of these barnacles, in particular on a marine turtle shell, remain challenging. This precludes the conduction of further manipulative experiments in the laboratory.

Nevertheless, a generalist approach can be beneficial for a marine biofouler in successful settlement on various hosts as in the *Chelonibia* barnacles. We postulate that the fitness/survival of *C. testudinaria*/*C. patula* is increases by the generalist approach as sea turtles are most likely not commonly encountered, and phenotypic plasticity can likely facilitate adaptations of the life on different hosts with contrasting life-styles. The observed morphological divergence is driven by environmental response at the present stage. Whether genetic assimilation (or genetic accommodation, see review of Waddington’s theory in [50]) would occur would depend on the interplay of various environmental factors, and thereby the relative cost of phenotypic plasticity and adaptive divergence. It might be possible that the single outlier locus detected represents early genetic changes following the initial adaptation by phenotypic plasticity. Hence speciation remains at early phase in the case of *Chelonibia.* Surely, these are just some possible outcomes in the future and phenotypic plasticity is the main player in the adaptation of *Chelonibia* in the current stage. Future study in transcriptomic profile during the development of different morphs settling on distinct hosts and more detailed outlier loci mining would be fruitful to explore the genetic basis of the observed plasticity and the potential of speciation, which would enhance our understanding in the interplay between phenotypic plasticity and adaptive divergence in the nature.

### Conclusion

Based on both morphological and molecular evidence, we propose that *Chelonibia testudinaria* and *C. patula* from SE Asia and Taiwan are conspecific and belong to the western Pacific population of *C. testudinaria* identified by Rawson et al [29]. The two taxa possess significant differentiation in shell morphology, and differential selection on the genome is inferred to be insignificant. Accordingly, the different shell morphs are believed to adapt to different hosts through developmental phenotypic plasticity. The survival of *C. testudinaria*/*C. patula* is postulated to be increased through the generalist approach. On the other hand, the high plasticity in morphology possibly improves the fitness of the species toward optimal in heterogeneous environments imposed by the broad host range and enables it to be a successful epibiotic biofouler. The present study provides valuable knowledge about adaptation and evolution of symbiotic fauna in the marine realm.

## Materials and Methods

### Sample Collection and Morphological Investigation

A total of 79 individuals of *Chelonibia patula* from various benthic crustaceans (*C. patula* are mainly found on crustaceans, see [10]) in Taiwan, Hong Kong, Singapore and Kuching in Malaysian Borneo and 25 *Chelonibia testudinaria* from marine turtles in Taiwan were collected (Figure 1D, Appendix S1). Barnacles on crustaceans are collected from local live seafood markets selling local catches of the fishermen from nearby waters and the species collected are not under protection nor need permit for collection. Barnacles on marine turtles are collected by Prof. I-Jiunn Cheng in Taiwan under the permit offered by the Taitung County Government, Republic of China. All *Chelonibia patula* and *C. testudinaria* collected were dissected for morphological analysis. Shell parameters including shell length and orifice length along the rostral-carinal axis, maximum shell thickness, maximum shell height, length of all margins in the scutum and tergum (Figure 1E) and the total length of the exopods of the left cirri IV, V and VI (from first segment after the basipod to the terminal segment; Figure 1F) were measured using digital vernier calipers (±0.1 mm). Since the size of *C. testudinaria* and *C. patula* collected were different (mean shell length *C. patula*: 9.4±3.8 mm, *C. testudinaria*: 38±17 mm), all the morphological parameters measured (except shell length) was divided by the shell length before subsequent analysis to reduce the error due to size differences between samples. Due to great size differences in body sizes between the barnacles on crustaceans and on turtles, absolute value on morphological parameters was not used for statistical analysis as the resulted variations between the two species may merely due to size but not on the morphological differences of the species. Variations in the morphological parameters were analyzed using multivariate analysis (PRIMER 6, Plymouth Routine in Multivariate Analysis; [51]). A similarity matrix was calculated between the *Chelonibia* samples using Euclidean distance. Non-metric multidimensional scaling (nMDS) was conducted to generate two dimensional plots of the scutum and tergum geometry between species from different geographical locations. Analysis of similarity (ANOSIM) was conducted to test the differences in opercular dimension between species and SIMPER (Similarity Percentage) analysis was used to detect the significant discriminating opercular diameters.

The somatic body was dissected and the arthropodal characters (mouth parts and the setal types in cirri I-VI, which are important taxonomic characters) were examined under scanning electron microscopy (SEM, preparation of samples for SEM following [22]). Setal definition followed [52].

### DNA Extraction and Sequencing of the Mitochondrial Markers

Isolation and purification of genomic DNA were conducted according to [22]. All the specimens were subjected to PCR for the mitochondrial COI, 12S and 16S rRNA gene segments. The PCR profile was as follows: initial 2.5 min denaturation at 95°C, 30 cycles of 30 s at 96°C, 30 s at various annealing temperatures (COI: 48°C, 12S and 16S: 52°C), 1 min at 72°C and final 3 min extension at 72°C. The primers used were LCO1490/HCO2198 for COI [53], those of [54] for 12S rRNA, and 1471/1472 for 16S rRNA [55]. The amplicons were sent to a commercial company (Genomics BioSci. and Tech., Taiwan) for purification and automated sequencing. Sequences obtained (refer to Appendix S1 for GenBank accession nos.) were visually edited and aligned using MEGA version 4 [56]. Both missing data and gaps were treated as missing data and excluded from analysis.

### Statistical Analysis on Mitochondrial Markers

To compare the COI sequences obtained from this study with those of *Chelonibia testudinaria* from the world’s oceans reported by [29], a maximum likelihood (ML) tree was constructed using RAxML-HPC Blackbox [57] through the online server Cyberinfrastructure for Phylogenetic Research (CIPRES; http://www.phylo.org; conducted on 9 Mar. 2011), while a Bayesian inference (BI) tree was inferred by the program MrBayes v.3.12 [58]. Default options (1000 bootstrap replicates for significance of branching) were used for ML tree construction. Transversional model (TVM) with proportion of invariable site (+I) and rate variation among site (+G) was estimated to be the optimal substitution model from Akaike information criterion by jModelTest version 0.1.1 [59]. This information was used to specify the *a priori* parameters in BI analysis, in which two Markov-chain-Monte-Carlo (MCMC) searches with random starting points were conducted in 10,000,000 generations; trees were sampled every 10,000 generations. The burn-in value was set to retain the last three-quarter of the sampling trees, from which the posterior possibility was calculated to illustrate the statistical support for nodes. Five haphazardly chosen sequences from each of the five sampling populations of *C. testudinaria* in [29] (GenBank accession nos.: AY174312-16, 24-28, 34-8, 42-46, 58-62) were added for tree construction, with three sequences of *C. caretta* (FJ385728-30) used as the outgroup. The sequences were trimmed to fit the shortest sequence length (*C. caretta*; 525 bp) analyzed in the alignment.

To infer the intraspecific population structure, haplotype networks of the three mitochondrial markers were generated by TCS version1.13 [60]. The haplotype and nucleotide diversities, as well as the Kimura’s two-parameter (K2P) distance [61], were calculated for the three markers using Arlequin version 3.1 [62] and MEGA, respectively. Unpaired t-test (http://www.graphpad.com/quickcalcs/ttest1.cfm?Format=SD) was used to test whether the diversities between the two species were significantly different. Locus-by-locus analysis of molecular variance (AMOVA), which was suggested to better accommodate any effect of missing data, was performed to demonstrate the partitioning of genetic variance among populations and between the two species, and to test if the partition is statistically significant through 10,000 permutations using Arlequin.

### Genotyping Using Amplified Fragment Length Polymorphism (AFLP)

Other than mitochondrial genealogical markers, the genome-wide genotyping method amplified fragment length polymorphism (AFLP) was used to elucidate the relationship between *C. patula* and *C. testudinaria* and determine whether there are loci under selection.

The quality of the genomic DNA of each specimen was first checked using agarose gel electrophoresis to ensure there was a clear, intact band (nuclear genome) near the wells. The AFLP profile of specimens with good DNA quality (76 samples) was generated according to [26]. Based on preliminary result, selective amplifications were conducted with the three primer combinations E_AGA/M_CTA, E_ACC/M_CTT and E_AAG/M_CTT, in which the M (Mse) primers were 5′ labeled with florescent FAM5, NED and VIC respectively. The products of the selective PCR were then sent to a commercial company (Techdragon, Hong Kong) for Genscan (fragment sizing), using florescent ROX-500 as the internal size standard.

Banding profiles of the specimens were visualized and scored using Genographer version 2.1.4 (http://sourceforge.net/projects/genographer). Viewing the gel under the intensity of 5, we scored the bands falling within 100 to 500 bp. The scoring threshold was set to 0.5 and the band width one (bp). A matrix of presence/absence binary data was then obtained. As recommended by [63] to have >5–10% subsamples to calculate the genotyping error rate [64], over 26% of samples (20 out of 76) was haphazardly chosen and genotyped twice in the present study. The average genotyping error rate of the loci estimated was 0.1, which was at the acceptable level (0.1) suggested by [63].

### Statistical Analysis on AFLP Dataset

AFLPsurv version 1.0 [65], which is an allelic-frequency-based method that utilizes the unbiased estimator of allelic frequency to assess genetic diversity (i.e. expected heterozygosity) [66], was used to estimate the pairwise relatedness coefficients between individuals of *C. patula* and *C. testudinaria*, and to test whether there is significant population differentiation between the species through 10,000 permutations, assuming Hardy-Weinberg genotypic proportion. The pairwise distances (i.e. 1- relatedness coefficients) among each genotyped specimen were graphically visualized through nMDS generated by PRIMER.

The AFLP candidate loci under selection pressure, which are the outlier loci demonstrating F_ST_ values significantly higher than overall mean values across the loci, were identified by BayeScan version 2.01 [67]. The false discovery rate (FDR) of this program was shown to be much lower than the other commonly used programs in detecting loci under selection and the multinomial Dirichlet model adopted in the program that allows for differential effective size and migration rate among populations was believed to be more ecologically realistic [67]. A locus-specific statistic, alpha, which is decomposed from the F_ST_ values that reflect the locus-wise difference of allele frequency, was used to infer the selection [67]. Departure from neutrality is assumed for the loci when alpha is significantly different from 0, whereas positive and negative alpha values indicate balancing or diversifying selection, respectively. This program adopts a Bayesian approach and uses reversible-jump MCMC to estimate the posterior probability of alpha, which was expressed as posterior odds (PO; the ratio of posterior probability of alpha in neutrality compared to that under selection). We carried out pilot runs, as default, to estimate the prior parameters and then ran 10 million iterations with thinning intervals of 1,000. The prior odds was set to 1 (the probability for the neutral model to happen is equal to that of the model with selection) so that the posterior odds generated could be directly interpreted as Bayes Factors. The first-quarter sampled data (2.5 million iterations) were discarded as burnin, and the remaining data were then used for the calculation of posterior odds. Graphical illustrations of the results were done using R-package [68] as described in the user manual [69].

## Supporting Information

Appendix S1 Detail information of *Chelonibia patula* and *C. testudinaria* specimens and their respective GenBank accession nos. Refer to table 2 for the acronyms of the populations.
(DOC)
