# Supplementary material for: Host-Specific Phenotypic Plasticity of the Turtle Barnacle Chelonibia testudinaria: A Widespread Generalist Rather than a Specialist
Source: PLoS One. 2013 Mar 1;8(3):e57592. doi: 10.1371/journal.pone.0057592 (PMC3585910; doi:10.1371/journal.pone.0057592)
Supplement: Appendix S1 — Detail information of Chelonibia patula and C. testudinaria specimens and their respective GenBank accession nos. Refer to table 2 for the acronyms of the populations. (DOC) [file pone.0057592.s001.doc]

Appendix 1. Detail information of *Chelonibia patula* and *C. testudinaria* specimens and their respective GenBank accession nos. Refer to table 2 for the acronyms of the populations.

| Specimen | Host | Site and date of collection (acronym of population) | Specimen genotyped by AFLP | GenBank accession number (in the order of COI; 16S; 12S) |
| --- | --- | --- | --- | --- |
| *C. patula* |  |  |  |  |
| Che_p_1 | Blue swimmer crab (*Portunus pelagicus*) | Western Market, Hong Kong, July 2003 (sc) | Che_p_1 | JF823661; JF823764; JF823866 |
| Che_p_2-4 | Sentinel crab (*Podophthalmus vigi*l) | He Ping Dau, Taiwan, Sept. 2008 (tw) | - | JF823662-4; JF823765-7; JF823867-9 |
| Che_p_5-7 | Indo-Pacific swimming crab (*Charybdis hellerii*) | Yuenlin Taiwan, 13 Apr. 2009 (tw) | Che_p_5-7 | JF823665-7; JF823768-70; JF823870-2 |
| Che_p_8-13 | Mantis shrimp (*Harpiosquilla harpax*) | SaiKung Pier, Hong Kong, 22 June 2009 (sc) | Che_p_8,9,11-13 | JF823668-73; JF823771-6; JF823873-75* |
| Che_p_14-28 | Mud crab (*Scylla serrata*) | Pasar Tamu Dbku (Kuching Holiday Market), Malaysia, 11 Oct. 2009 (ma) | Che_p_15, 18, 22, 24-27 | JF823674-88; JF823777-91; JF823876-90 |
| Che_p_29-76 | Mud crab (*Scylla serrata*) | Jurong Fishery Port, Singapore, 08 Oct. 2009 (si) | Che_p_29-41, 43-70, 72-75 | JF823689-735*; JF823792-838*; JF823891-938 |
| Che_p_77-79 | Crab (Eucrate crenata) | Guangxi, China; July 2008 (sc) | - | JF823736-8; JF823839-41; JF823939-41 |
| *C. testudinaria* |  |  |  |  |
| Che_t_1 | Loggerhead sea turtle (*Caretta caretta*) | Dong Ao, Lanyu, Taiwan; Jan 2006 (tw) | - | JF823739; JF823842; JF823942 |
| Che_t_2,3,5-7 | Hawksbill sea turtle (*Eretmochelys imbricata*) | Dong Ao, Lanyu, Taiwan; 17 Jan. 2006 (tw) | - | JF823740-1,3-5;  JF823843-4, 6-8; JF823943-4, 6-8 |
| Che_t_4 | Sea turtle, no species records | Lin-yuen, Kaoshiung, Taiwan; 17 Apr. 2003 (tw) | Che_t_4 | JF823742; JF823845; JF823945 |
| Che_t_8-10 | Loggerhead sea turtle (*Caretta caretta*) | Dong Ao, Lanyu, Taiwan; Jan 2006 (tw) | - | JF823746-8; JF823849-51; JF823949-51; |
| Che_t_11-12 | Loggerhead sea turtle (*Caretta caretta*) | Dong Ao, Lanyu, Taiwan; 14 Dec. 2009 | Che_t_11-12 | JF823749-50; JF823852-3; JF823952-3 |
| Che_t_13-17 | Green sea turtle (*Chelonia mydas*) | Dong Ao, Taiwan; 24 Dec. 2009 (tw) | Che_t_14-17 | JF823751-5; JF823854-8; JF823954-8 |
| Che_t_18-20 | Loggerhead sea turtle (*Caretta caretta*) | Dong Ao, Lanyu, Taiwan; (tw) | Che_t_18-20 | JF823756-8; JF823859-60*; JF823959-61 |
| Che_t_21-25 | Green sea turtle (*Chelonia mydas*) | Caoli fishing port, Taiwan; 17 May. 2010 (tw) | Che_t_21-25 | JF823759-63; JF823861-5; JF823862-6 |

* Missing sequences due to unsuccessful PCR amplification: Che_p_8 (12S), 10 (12S), 11 (12S), 76 (COI, 16S) and Che_t_19 (16S)
